# Supplementary material for: Enhancing Escherichia coli abiotic stress resistance through ornithine lipid formation
Source: Appl Microbiol Biotechnol. 2024 Apr 8;108:288. doi: 10.1007/s00253-024-13130-5 (PMC11001654; doi:10.1007/s00253-024-13130-5)
Supplement: Supplementary file 1 [file 253_2024_13130_MOESM1_ESM.pdf]

Supplementary material

Submitted to: Applied Microbiology and Biotechnology

AMBB-D-23-00392R1

Enhancing *Escherichia coli* Abiotic Stress Resistance through Ornithine Lipid Formation

Leidy Patricia Bedoya-Pérez[1], Alejandro Aguilar-Vera[1], Mishael Sánchez-Pérez, José Utrilla[1*], Christian Sohlenkamp[1*]

[1]Centro de Ciencias Genómicas, Universidad Nacional Autónoma de México, Av. Universidad s/n Col. Chamilpa, C.P. 62210, Cuernavaca, Mor., México.

*Corresponding authors

Name: José Utrilla: utrilla@ccg.unam.mx

Name: Christian Sohlenkamp: chsohlen@ccg.unam.mx

Address: Centro de Ciencias Genómicas, Universidad Nacional Autónoma de México, Av. Universidad s/n, Col. Chamilpa, C.P. 62210, Cuernavaca, Mor., México.

Supplementary material

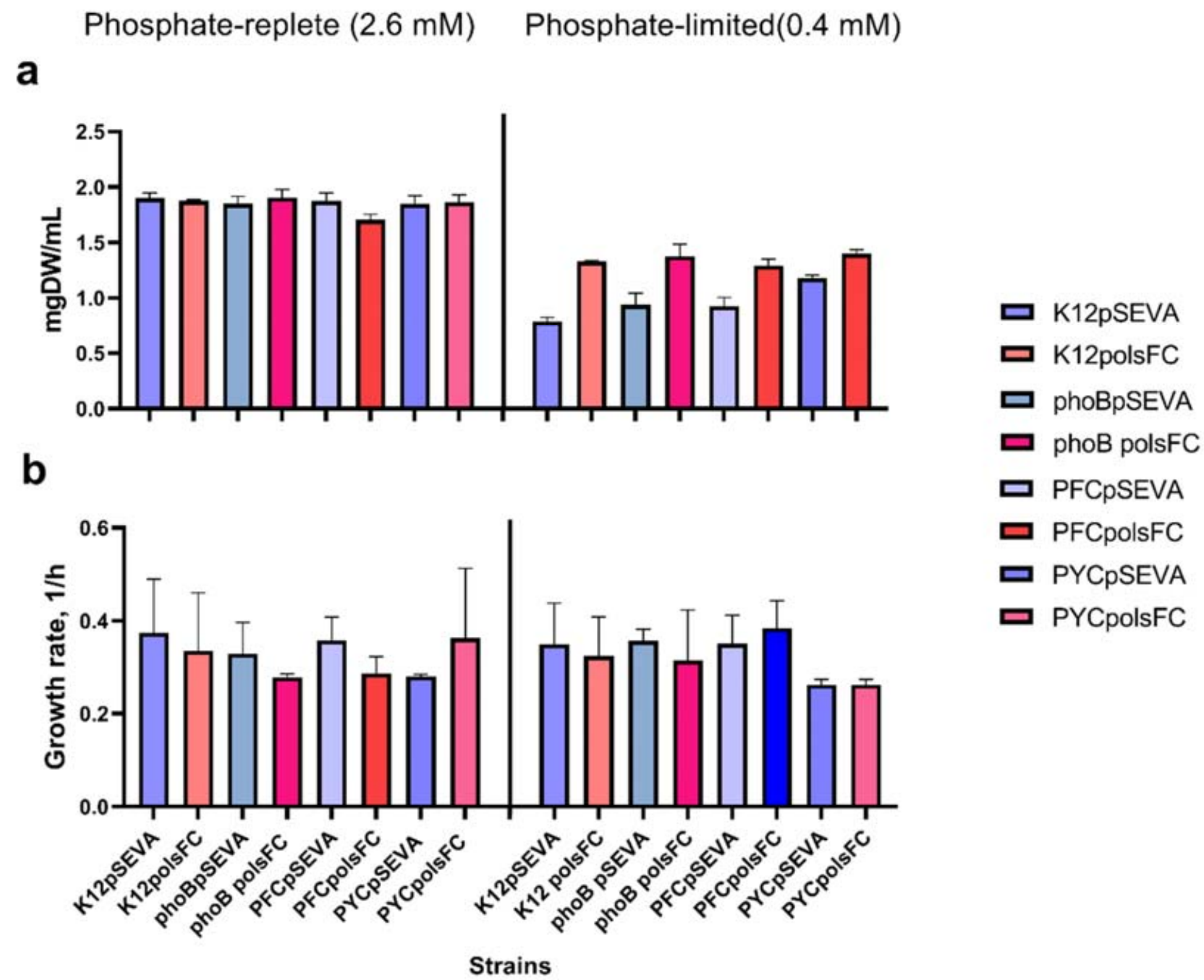

S1. Synthesis of ornithine lipids (uOLs and OLs-OH) in *E. coli* increases biomass production under phosphate-limited conditions but does not affect the growth rate. (a) dry weight concentration (mgDW/mL) and (b) maximum specific growth rate are shown. Cells were grown in 96-well plates for 24 hours at pH 7.4 in MOPS/glucose medium without phosphate limitation (2.6 mM) and with phosphate limited (0.4 mM). Each strain was grown harboring the empty control plasmid or the plasmid containing *olsFC*. The data represent the average of three independent experiments. The error bars indicate the standard deviation using a two-tailed unpaired student's t-test.

Biomass yield Phosphate-Limited

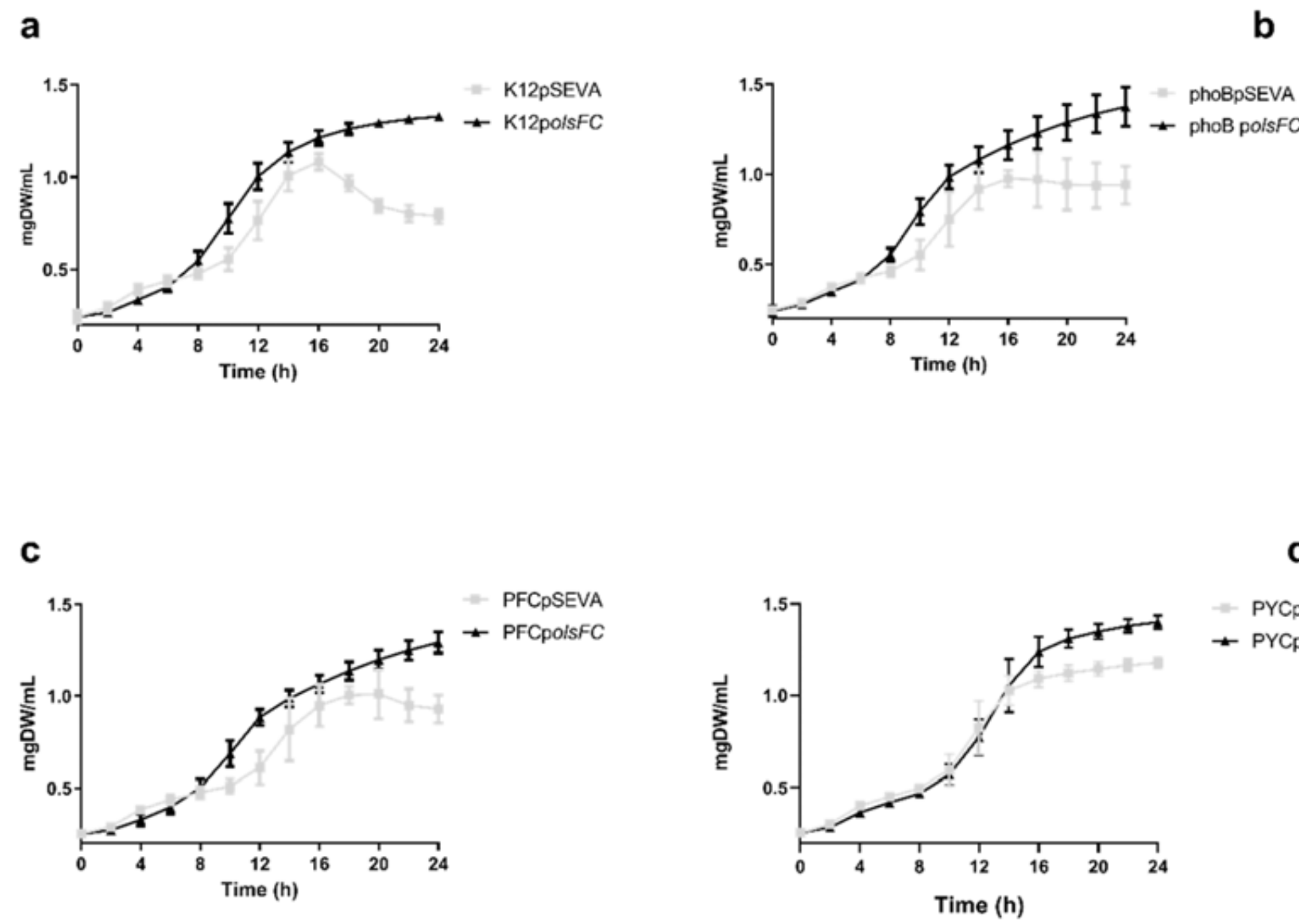

S2. **The presence of ornithine lipids (uOLs and OLs-OH) in *E. coli* strains improves their growth characteristics under phosphate limitation.** Growth curves of *E. coli* K12 (a), *E. coli* mutant *phoB* (b), *E. coli* triple mutant PFC (c), *E. coli* triple mutant PYC (d), in MOPS/glucose medium with 0.4 mM phosphate at pH 7.4.

Low pH

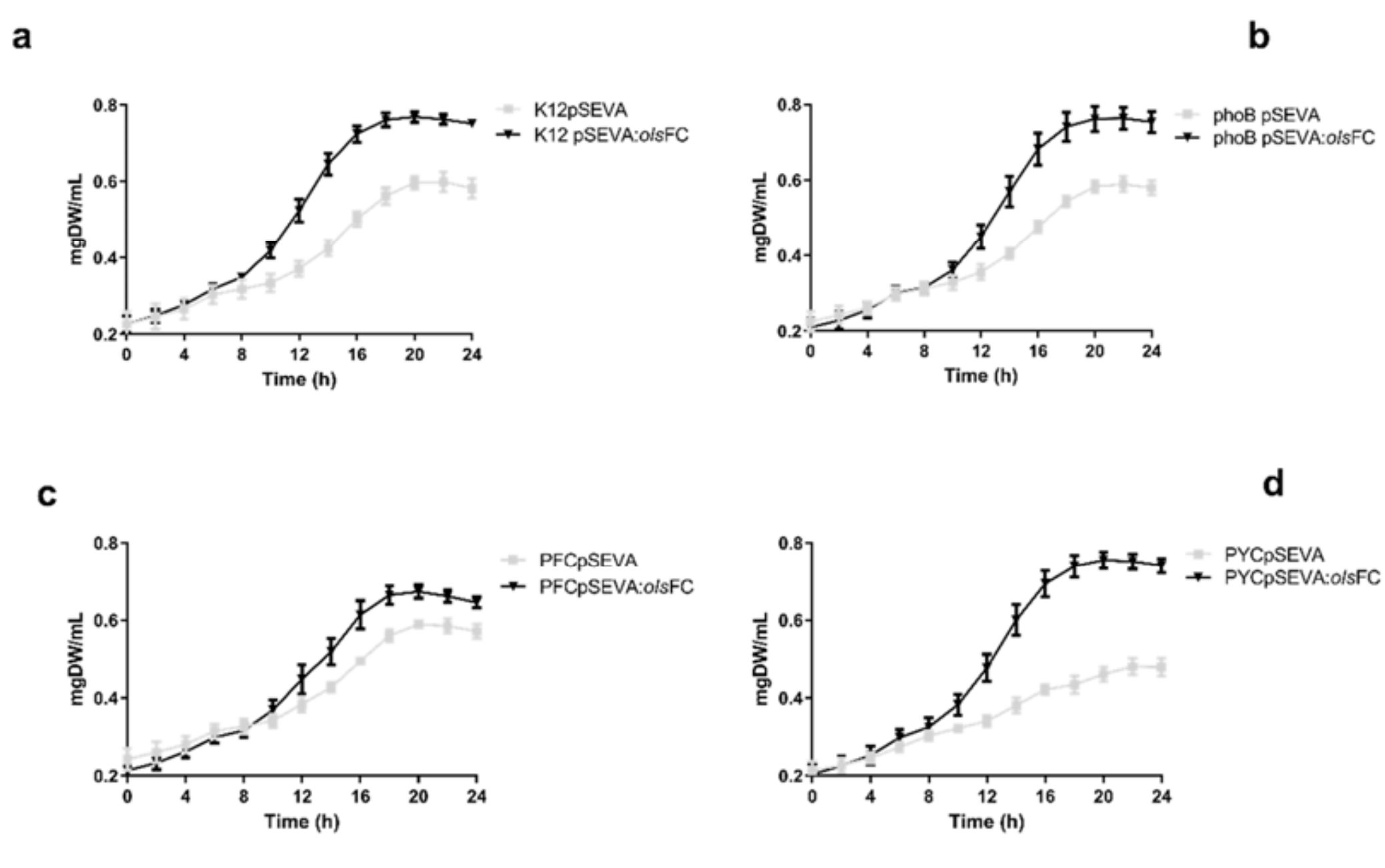

S3. **The presence of ornithine lipids (uOLs and OLs-OH) in *E. coli* strains improves their growth characteristics at pH 5.8.** Growth curves of *E. coli* K12 (a), *E. coli* mutant *phoB* (b), *E. coli* triple mutant PFC (c), *E. coli* triple mutant PYC (d) in MES/glucose medium with 2.6 mM phosphate at pH 5.8.

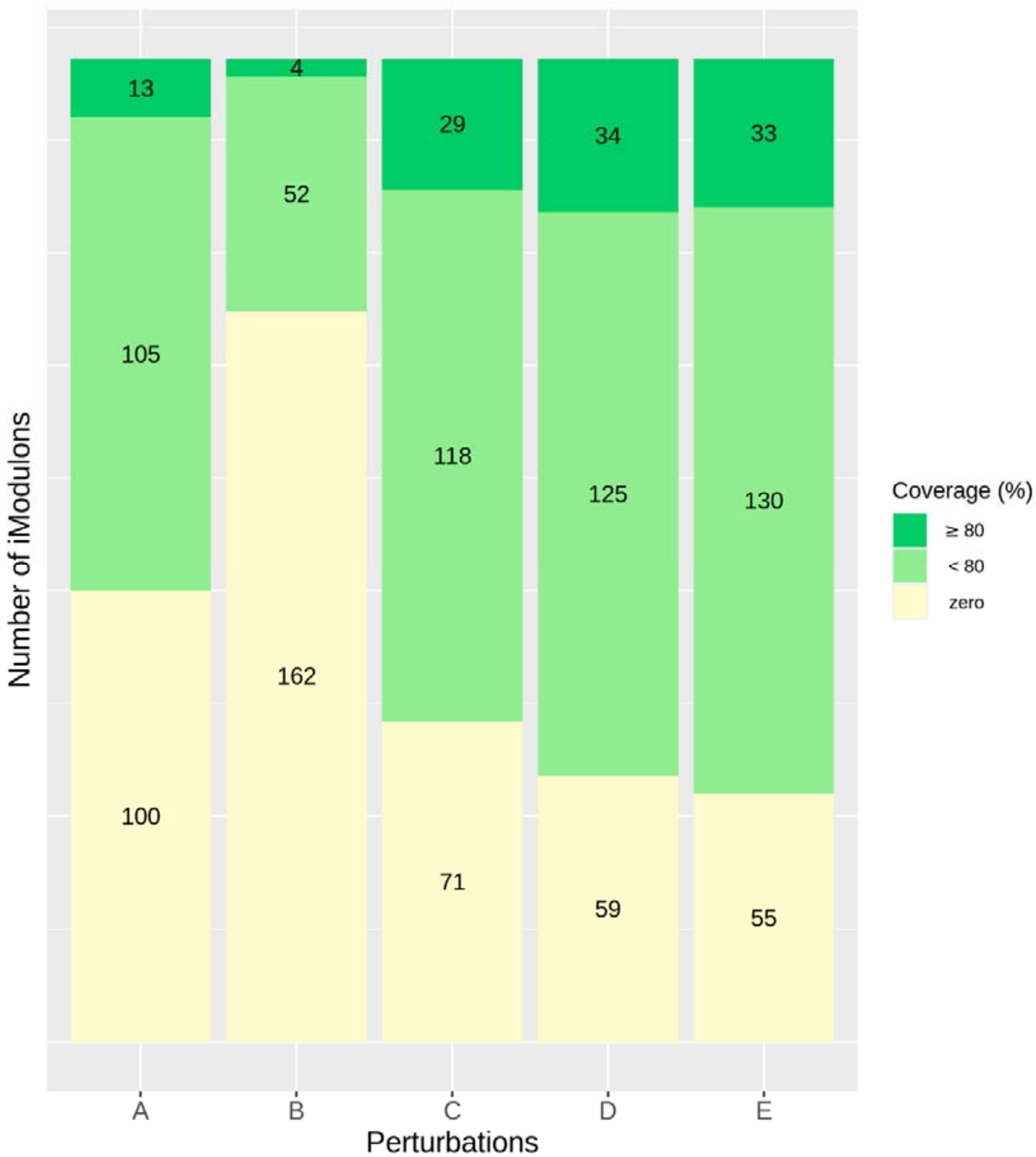

S4. Numbers of iModulon in the different perturbations. This classification consists in the clustering of genes that share an independent modulated signal. IModulons coverage is represented in dark green when is ≥80% of genes, light green <80% and beige when no changes in expression were observed.

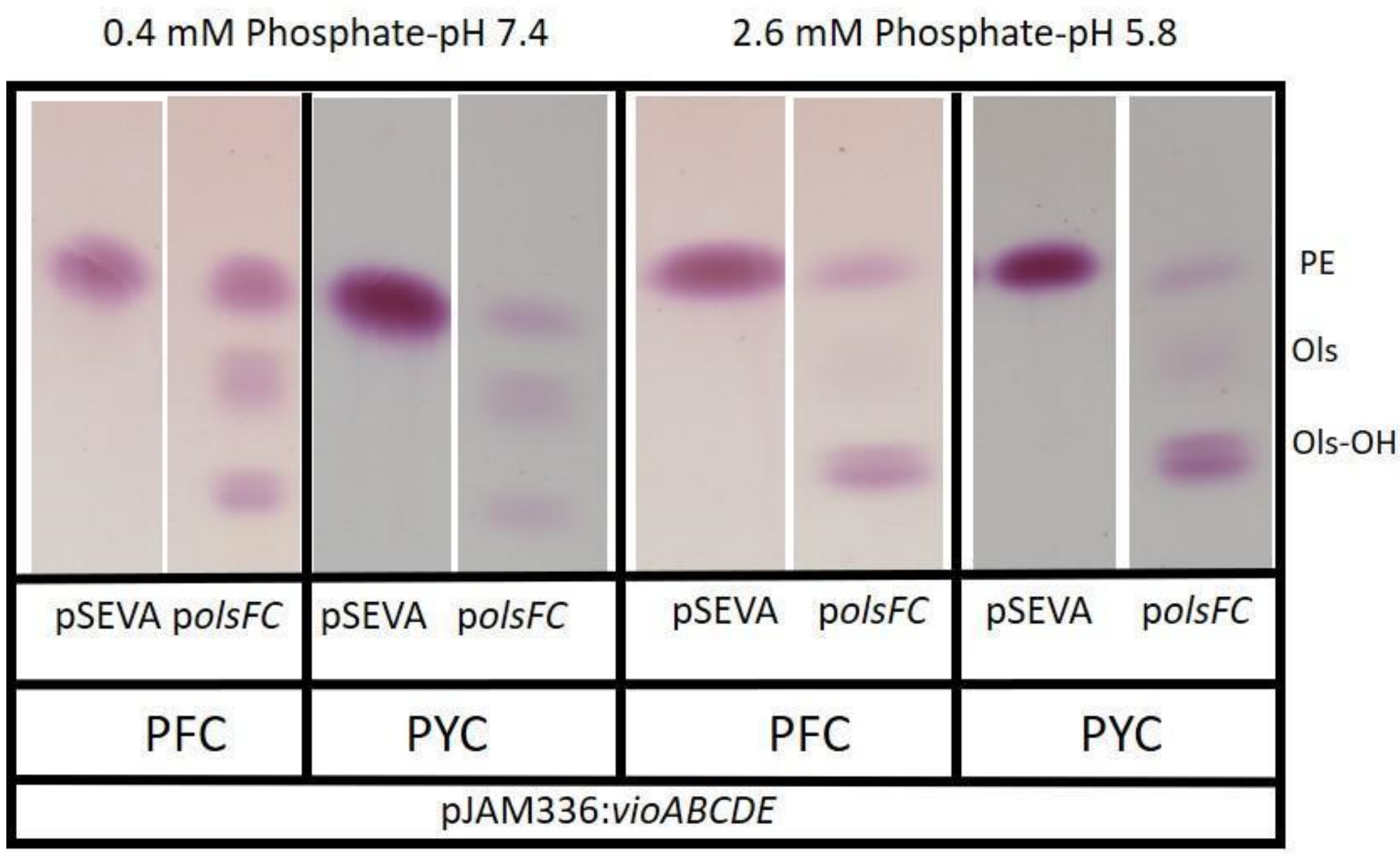

S5. *E. coli* strains forming OLs (uOLs and OLs-OH) and synthesizing violacein. Thin layer chromatography (TLC) analysis of total lipids from *E. coli* strains forming OLs and harboring the violacein synthesis pathway. The TLC plate was stained with 0.2% ninhydrin to detect primary amine-containing lipids which are in this case PE, OL, and OLs-OH. All strains analyzed for their lipid composition harbored the plasmid pJAM336:*vioABCDE* in addition to a second plasmid. Strains PFC and PYC with pSEVA (empty) and p*olsFC* with pJAM336:*vioABCDE* grown in MOPS medium with 0.4 mM phosphate and pH 7.4 (left) and MES 2.6 mM and pH 5.8 (right). PE: phosphatidylethanolamine; uOL: unmodified ornithine lipid; OLs-OH: hydroxylated ornithine lipid.

Table S1. Strains and plasmids used in this study

STRAINS	GENOTYPE OR DESCRIPTION	REFERENCE
PFC	*E. coli* K12BW25113 with mutations in *phoB, yedW, cusR*	Lastiri-Pancardo *et al.*, 2020
PYC	*E. coli* K12BW25113 with mutations in *phoB, fhlC y cueR*	Lastiri-Pancardo *et al.*, 2020
PhoB	*E. coli* K12BW25113 with mutation in *phoB*	KEIO collection
E. coli K12 BW25113	*E. coli* K12BW25113 wild-type strain	Laboratory stock
E. coli K12 BW25113pSEVA	*E. coli* K12BW25113 wild-type strain harboring pSEVA631 Gm^{r}	This study
phoBpSEVA	*phoB* strain harboring pSEVA631 Gm^{r}	This study
PYCpSEVA	PYC strain harboring pSEVA631 Gm^{r}	This study
PFCpSEVA	PFC strain harboring pSEVA631 Gm^{r}	This study
PFCp*olsFC*	PFC strain harboring pSEVA:*olsFC* Amp^{r}	This study
PYCp*olsFC*	PYC strain harboring pSEVA:*olsFC* Amp^{r}	This study
PhoBp*olsFC*	*pho* strain harboring pSEVA:*olsFC* Amp^{r}	This study
K12p*olsFC*	*E. coli* K12BW25113 wild-type strain harboring pSEVA:*olsFC* Amp^{r}	This study
PYCpSEVA:*vio*	PYC strain harboring pSEVA Amp^{r} and pAJM.336*pvioABCDE* Km^{r}	This study
PFCpSEVA:*vio*	PFC strain harboring pSEVA Amp^{r} and pAJM.336*pvioABCDE* Km^{r}	This study
PYC*polsFC*:*vio*	PYC strain harboring pSEVA:*olsFC* Amp^{r} and pAJM.336*pvioABCDE* Km^{r}	This study
PFC*polsFC*:*vio*	PFC strain harboring pSEVA:*olsFC* Amp^{r} and pAJM.336*pvioABCDE* Km^{r}	This study
pCCS98	*olsC* of *R. tropici* in pET9a	Vences *et al.* 2011
pET9a-2569	*olsF2569* of *Serratia proteomaculans* in pET9a	Vences *et al.* 2015
pSEVA63-Hvio	*vioABCDE* operon of *Chromobacterium* in pSEVA631	Darlintong *et al.* 2018

Table S2. Oligonucleotides used in this study. All sequences are written in 5’ to 3’ direction.

**pSEVA*olsF***	
pSEVA_*olsF* REV	AGAGAGTCCAAGCTAAACATCTAGTATTTCTCCTCTTTCTCTAGTATTTAT
pSEVA_ *olsF* FWD	AGGACACCTGTATAGAGTAATACTAGAGCCAGGCATCGCA
Spro_2569*olsF* FWD	AGAAAGAGGAGAAATACTAGATGTTTAGCTTGGACTCTCT
Spro_2569*olsF* REV	TGCGATGCCTGGCTCTAGTATTACTCTATACAGGTGTCCTGT
**pSEVA*olsFC***	
pSEVA_*olsFC* FWD	GAAACCGCCCGAAGCCCTGACGCAAAAAACCCCGCTTCGG
pSEVA_*olsFC* REV	TCCGTCATTCCAAACCTCCTATGCCTGGCTCTAGTATTACTCTATAC
pCCS98_*olsC* FWD	GTAATACTAGAGCCAGGCATAGGAGGTTTGGAATGACGGAGA
pCCS98_*olsC* REV	CCGAAGCGGGGTTTTTTGCGTCAGGGCTTCGGGCGGTTTC
**pAJM.336:*vioABCDE***	
pAJM.336_*vioE* FWD	TTTTTTCTGCCTCGTGATACGCCTACTCGGTACCAAATTCCAGAAAAGAG
pAJM.336_*vioA* REV	TGCAGATATCGGAAGAATGCTTCATCTAGTATTTCCCCTCTTTCTCTAGTATTAAAC
vioA FWD	ACTAGAGAAAGAGGGGAAATACTAGATGAAGCATTCTTCCGATATCTGCATTGTCG
vioA REV	TAGTATTTCTCCTCTTTCTCTAGTATAGAGGATCCCCGGGTACCGAGCTC
vioB_E FWD	GAGCTCGGTACCCGGGGATCCTCTATACTAGAGAAAGAGGAGAAATACTAGATGAG
vioB_E REV	CTCTTTTCTGGAATTTGGTACCGAGTAGGCGTATCACGAGGCAGAAAAAA
